# Analysis of anti-tuberculosis drug resistance and sociodemographic and clinical aspects of patients admitted in a referral hospital

**DOI:** 10.31744/einstein_journal/2020AO4620

**Published:** 2019-10-22

**Authors:** Camilla Resende Bonin, Romário Costa Fochat, Isabel Cristina Gonçalves Leite, Thamiris Vilela Pereira, Marina de Oliveira Fajardo, Carmen Perches Gomide Pinto, Raquel Leite Macedo, Marcio Roberto Silva, Pillar Pace Lacerda Menezes, Nilma Maria José Mendes de Araújo, Ronaldo Rodrigues da Costa

**Affiliations:** 1 Universidade Federal de Juiz de Fora, Juiz de Fora, MG, Brazil.; 2 Hospital Universitário, Universidade Federal de Juiz de Fora, Juiz de Fora, MG, Brazil.; 3 Empresa Brasileira de Pesquisa Agropecuária, Brasília, DF, Brazil.; 4 Fundação Hospitalar do Estado de Minas Gerais, Belo Horizonte, MG, Brazil.

**Keywords:** Tuberculosis, Tuberculosis, multidrug-resistant, Mycobacterium tuberculosis, HIV, Epidemiology

## Abstract

**Objective:**

To determine the occurrence of anti-tuberculosis drug resistance and its association with sociodemographic and clinical characteristics of patients in a referral hospital.

**Methods:**

This was a cross-sectional study based on data from patients who had mycobacterial culture identified and defined antimicrobials sensitivity profile (June 2014 to February 2016). The descriptive statistical analysis and Fisher’s exact test were used to compare proportions.

**Results:**

The study included 104 patients who had positive results for *Mycobacterium tuberculosis* . Bacilloscopy had high positivity (93.3%). A total of 15 patients (14.4%) had resistant strains and six (5.6%) multidrug-resistant. The sociodemographic and clinical characteristics were not related with resistance.

**Conclusion:**

This study contributed to further the understandings about the tuberculosis patients’ profile, the study also served as a tool for development of specific public policies. Patients diagnosed with resistant tuberculosis must be under greater supervision.

## INTRODUCTION

Tuberculosis (TB) is an ancient infectious disease that is recognized as a global public health problem, and constitutes a challenge to be faced by all countries.^( 1 , 2 )^ The association of correct diagnosis and adequate treatment can contribute to the cure of individuals with TB. However, this infection caused by the *Mycobacterium tuberculosis* bacillus is one of the leading death causes among adults by infectious diseases. In 2016, according to estimations of the World Health Organization (WHO), 10.4 million new cases of TB appeared worldwide, and the disease caused approximately 1.6 million deaths.^( 1 )^

Brazil is part of the list of priority countries issued by the WHO and has a great impact in the TB global scenario with 82.676 notified cases in 2016, an incidence of 42.0/100 thousand of inhabitants with the disease, and a death rate due to TB of 3.5 deaths/100 thousand of inhabitants).^( 1 )^

Although Brazil has achieved the proposals for millennium development goals (decreasing of TB incidence by 2015 and reduction of 50.0% of the disease prevalence rates, and its mortality rates compared with 1990),^( 3 )^the country has huge challenges to be overcome in the addressing of new strategies to eradicate TB by 2050.^( 4 )^ The agenda continues with demands related with prevention, early effective diagnoses, adherence to treatment, and the emerging problem of increasing in resistance to available drugs.^( 1 , 5 )^

In Brazil, the Tuberculosis Special Treatment Cases Information System (SITE-TB - *Sistema de Informação de Tratamentos Especiais de Tuberculose* ) is the main surveillance tool for TB cases that require special treatment, such as drug-resistant TB. The medication for treatment of resistant TB is distributed in referral centers after approval of the SITE-TB. In 2016, according to data collected in the Brazilian Information System on Notifiable Diseases (SINAN - *Sistema de Informação de Agravos de Notificação* ), 1,044 drug-resistant TB cases were reported in the country, whereas in the SITE-TB, in the same year, 752 news drug-resistant TB cases were registered. This information, even considering the limitations inherent to the systems, is a concern, because it suggests that many patients do not conduct adequate follow-up.^( 6 )^

The controlling and management of program to combat resistant TB are more difficult compared with tasks required for the program of patients who have sensibility to treatment.^( 7 )^ Risk factors, such as nutrition deficiency, smoking, HIV/AIDS infection, *diabetes mellitus* , poor ventilated environment, and densely occupied spaces are commonly reported in the published literature as responsible for maintenance and worsening of TB.^( 1 , 4 , 5 , 8 - 10 )^

Epidemiological features and disease control range depend on the geographic region and they are influenced by socioeconomical, educational and political factors. In the Zona da Mata region, the city of Juiz de Fora, Minas Gerais state, Brazil is considered a strategic municipality concerning the battle against TB.^( 11 , 12 )^ Identifying resistant TB cases and understanding the profile of individuals affected by this condition can provide evidences for clinical management of patients and for elaboration of more effective public policies.^( 13 )^ In addition, to conduct further studies on TB is one of the pillars highlighted by the WHO in the journey to achieve the new goals to eradicate this disease.^( 14 )^

## OBJECTIVE

To determine drug-resistant tuberculosis prevalence among patients diagnosed with this condition in a public hospital. To investigate among these patients the association of their condition with socidemographic and clinical features.

## METHODS

The *Hospital Regional Joao Penido* is located in the Zona da Mata region in the municipality of Juiz de Fora, Minas Gerais. This is a public hospital that provides care for users of the Brazilian Public Health System (SUS - *Sistema Único de Saúde* ) in secondary and tertiary levels of health care. The hospital deliveries care in Southeast macroregion for, approximately, a population of 1.5 million people. The hospital has an emergency room and outpatient’s units for elective consultations of number of specialties. During our study, the hospital was working with capacity of tertiary care covering 212 beds. This hospital is considered referral for treatment of TB.^( 15 )^

This was a cross-sectional study that included data collected from the Integrated Hospital Management System (SIGH - *Sistema Integrado de Gestão Hospitalar* ), an integrated system used by the hospital that allows health professional from the *Fundação Hospitalar do Estado de Minas Gerais* , who works in the hospital, to access users’ data, clinical information, procedures and exams previously performed in any unit of the foundation.

A retrospective analysis was conducted including records of the laboratory of clinical analysis of the hospital from June 2014 to February 2016. As inclusion criteria, we considered all patients with TB, regardless of age, who had positive culture for mycobacterium, specimen identification and sensibility profile to preconized antimicrobial agents.

Patients who had contaminated samples or inconclusive results for specimen or antimicrobial susceptibility testing (AST) were not considered. Positive cultures of isolated mycobacterial in the hospital were forwarded to *Fundação Ezequiel Dias,* where identification and AST were processed.

Drugs tested were streptomycin (S), isoniazid (H), rifampicin (R), ethambutol (E), and pyrazinamide (Z). Results interpretations of AST were performed based on WHO recommendations, organized by the Brazil Ministry of Health: monoresistance (resistance to one medicine only), multiresistance (simultaneous resistance by at least R and H), poliresistance (resistance to two or more drugs, except by previous association) and extensive resistance (resistance to R and H and, simultaneously, to fluoroquinolone and to second line injectable medicine).^( 16 )^

We collected information of individuals from SIGH regarding sex, age, type of residential, baciloscopy result (Ziehl-Neelsen coloration), clinical form of TB, coinfection by HIV, anemia, and use of tobacco products, alcohol, and illicit drugs. These information were validated by two evaluators at different times.

In case of result from sensible and other resistant culture (different samples) to a single patient, we provided priority to the resistance situation to categorize the individual. The Fisher exact test was used for data correlation with occurrence of resistance cases; the significance level adopted was 0.05. All calculations, including descriptive statistical analysis, were performed using the Statistical Package for the Social Sciences software (SPSS, Versão 14.0).

The protocol of the present study was approved by the Human Research Ethics Committee where this study was conducted, statement number 732.595/2014, CAAE: 34039214.5.0000.5119.

## RESULTS

We included 104 patients who had positive result for mycobacterial culture, identification of bacillus specimen and respective characterization of resistant profile. This sample was, predominantly, composed by men (79; 76.0%). Participants’ age ranged from 17 to 77 years (mean±standard deviation of 40.2±15.2 years). The majority of participants were young, they age ranged from 15 to 44 years (63; 60.6%).

Clinical diagnosis of pulmonary TB was presented by almost all individuals (100; 96.2%), and was verified only 2 cases (1.9%) from miliary TB, one (1.0%) from ganglionic TB, and one (1.0%) from pleural TB. Bacilloscopy reached high positivity index for this population (97; 93.3%). The isolated bacillus in all cultures was *Mycobacterium tuberculosis* , and was reported 15 cases (14.4%) of some drug-resistance used in treatment of the disease. We observed five cases of monoresistance (4.9%), six of multiresistance (5.6%) and four of poliresistance (3.9%) ( Table 1 ).

**Table 1:** Results from sensibility to antimicrobial of isolated mycobacterium of patients diagnosed with tuberculosis

Test	n (%)
Antimicrobial susceptibility testing	
Sensible	89 (85.6)
Resistant	15 (14.4)
Monoresistance	
Streptomycin	3 (2.9)
Pyrazinamide	1 (1.0)
Isoniazid	1 (1.0)
Multiresistance	
Isoniazid + rifampicin	6 (5.6)
Poliresistance	
Isoniazid + streptomycin	3 (2.9)
Isoniazid + streptomycin + ethambutol	1 (1.0)

For this sample, we could not establish significant statistically association between occurrence of anti-tuberculosis drug resistance, sociodemographic and clinical features available for patients ( Table 2 ). However, among resistance cases, the most frequent occurred among men (11; 73.3%), aged 15 to 44 years (10; 66.7%), who lived in a house/apartment (10; 66.7%), were diagnosed with pulmonary TB (14; 93.3%), HIV negative (9; 60.0%), had anemia (10; 66.7%) and who reported use of alcohol, tobacco products and others illicit drugs (10; 66.7%).

**Table 2:** Distribution of sociodemographic and clinical data of patients diagnosed with tuberculosis in relation to anti-tuberculosis drugs sensibility profile

Features	Sensible	Resistant	p value	Totaln (%)
	n (%)*	n (%)^†^	n (%)^‡^	n (%)
Sex			0,511	
Male	68 (76.4)	11 (73.3)		79 (76.0)
Female	21 (23.6)	4 (26.7)		25 (24.0)
Age range (years)			0.413	
15-44	53 (59.5)	10 (66.7)		63 (60.6)
>45	36 (40.5)	5 (33.3)		41 (39.4)
Type of residential			0.449	
House/Apartment	69 (77.5)	10 (66.7)		79 (76.0)
Prisoner/homeless	15 (16.9)	3 (20.0)		18 (17.3)
Not reported	5 (5.6)	2 (13.3)		7 (6.7)
Clinical form			0.469	
Pulmonary	86 (96.6)	14 (93.3)		100 (96.2)
Extrapulmonary	3 (3.4)	1 (6.7)		4 (3.8)
HIV			0.635	
Yes	13 (14.6)	3 (20.0)		16 (15.4)
No	50 (56.2)	9 (60.0)		59 (56.7)
Not reported	26 (29.2)	3 (20.0)		29 (27.9)
Anemia			0.466	
Yes	41 (46.0)	10 (66.7)		51 (49.0)
No	39 (43.8)	4 (26.6)		43 (41.3)
Not reported	9 (10.2)	1 (6.7)		10 (9.6)
Use of tobacco, alcohol, and other drugs			0.147	
Yes	53 (59.6)	10 (66.7)		63 (60.6)
No	33 (37.1)	3 (20.0)		36 (34.6)
Not reported	3 (3.3)	2 (13.3)		5 (4.8)

Tested drugs: streptomycin, isoniazid, rifampicin, ethambutol, and pyrazinamide. ^*^ Individual group (n=89) with non-resistant tuberculosis; ^†^ individuals group (n=15) with resistant tuberculosis; ^‡^ Fischer’s exact test with significance level of 0.05.

## DISCUSSION

Our study found a high general prevalence of anti-tuberculosis drug resistance (14.4%). Of this prevalence, 4.9% was monoresistance, 5.6% multiresistance, and 3.9% poliresistance. General resistance was slightly higher than results reported by other studies conducted in North region of Sao Paulo and in Santa Catarina.^( 18 )^ In this later state, authors analyzed 406 cultures, including 48 (11.8%) resistant cases, whereas the research conducted in state of Sao Paulo identified 348 cultures in which 31 (8.9%) had some type of resistance. The resistance profile described in the state of Sao Paulo revealed a predominance of monoresistance (23/31) whereas in Santa Catarina, 23/48 cultures were multiresistances. In the studied hospital, cases of resistant TB are well-distributed within WHO classification with slightly predominance of multiresistant TB (6/15).

Monitoring of resistant mycobacterium, highlighted in the report The End TB Strategy,^( 14 )^ is one of the strategic actions established to reduce mortality and incidence of TB in all countries. Population studies have been conducted in different sites to improve the understanding of occurrence of this situation.^( 13 , 17 - 22 )^ The primary care is an ideal level to detect early disease, but tertiary heath care can be the most common entrance point for patients in the system.^( 17 )^ Because the hospital where this study was conducted is a referral center for TB treatment for large geographic area (Southeast macroregion, in which, approximately, an population of 1.5 million people is covered),^( 15 )^ this allow relevant epidemiological studies in local and national scenario.

Care for hospital population can be the hospital-based care for those who need more urgent care, or outpatient-based care for those who could be followed-up with periodic consultations. The hospital deliveries care for an heterogeneous population, receives patients with suspicions or clinical complications associated with TB, including risk groups such as homeless, prisoners and/or drug users. All patients with suspicion of pulmonary TB undergo sputum bacilloscopy and mycobacterial culture with sensibility test. The possibility of the hospital to attend the most severe cases or those who treatment have failure can underestimate the prevalence of resistant TB.

The predominance of men in our study population (76.0%) corroborates with international and national data.^( 1 , 10 , 23 - 25 )^ Viana et al.,^( 10 )^ conducted a national descriptive epidemiological population-based study, considering all notification in SINAN between January 2008 and December 2011. These authors registered 278,674 news cases reported in the analyzed period and higher prevalence of men (65.7%). Others studies reported prevalence of TB in men ranging from 65.0% to 85.0% in different regions of Brazil.^( 17 , 19 , 23 - 25 )^Studies conducted in the states of Sao Paulo,^( 17 )^ Goias^( 19 )^ and Amazonas^( 20 )^ have observed no significant association with sex between resistant and multiresistant TB occurrence.

Of the studied population, 60.6% were aged from 15 to 44 years. This result also agree with studies from Viana et al.,^( 10 )^ (53.9% of reported cases were individuals aged 20 to 44 years), and others.^( 17 , 19 , 23 , 24 )^ The economically active age range of population is constantly related with TB, and this impacts management of public expenses.^( 10 , 23 - 25 )^ The patient with resistant TB increases health services-related costs and can cause an economic overload once he/she may have reduced prognosis, require longer treatment time, present more collateral and toxic effects of medications and need greater control actions to avoid transmission.^( 20 , 21 , 26 )^ Studies by Pedro et al.,^( 17 )^ and Santos et al.,^( 19 )^did not show association between resistant TB and any specific age range either.

Previous studies have associated pulmonary form of TB to higher probability of drug resistance. In extrapulmonary TB, the number of bacillus is low compared with those observed in pulmonary cavity, therefore, chances of resistant bacillus existence before treatment are lower.^( 23 , 27 )^ In the study by Pedro et al.,^( 17 )^ the pulmonary form of TB was predominant in the population (96.2% of cases), however, there was not significant relation with resistance occurrence, as we observed in our studied patients (96.2% of pulmonary TB).

Pulmonary TB requires higher priority with control actions because this is a transmissible clinical form. For this reason, the treatment and break of the transmission cycle, to be effective, requires precise and rapid diagnosis.^( 1 , 4 , 8 )^ The bascilloscopy continues to be the chosen method, mainly in developing countries, because this exam is simple, rapid, has low cost and, when performed correctly, allow to identify more than 70.0% of pulmonary TB cases.^( 28 )^ The bacilloscopy performed in patients attended in our hospital reached high positivity (93.3% of samples), which allows early diagnosis and higher safety for disease control, in addition to provide data on quality standard of the service and guidelines for patients at the time of collection.

Tuberculosis bacteria are spread through the air, for this reason, closed rooms and/or in areas with reduced hygiene are favorable to continuity of the disease. The prison population, individuals who live in overcrowding conditions, or are homeless are considered risk groups for the development of the disease.^( 8 , 10 , 22 , 29 )^ The incidence of TB in prisons can exceeds 20 times of the incidence recorded for the population as a whole.^( 29 )^ Among homeless individuals, this incidence can be 50 to 60 times higher than the general population.^( 30 )^

Because our study includes the population assisted by the hospital where the study was conducted and is not limited to specific groups, our results may have higher limitation to compare data of the literature, because there are few studies with the same methodological model. We should highlight, however, that Pedro et al.,^( 17 )^ observed, in the State of Sao Paulo that resistance to anti-tuberculosis drugs was statistically significance lower in liberty-deprived individuals than nondetainees. In the study by Santos et al., which considered the State of Goias,^( 19 )^ seven individuals (5.3% of population from the sample) were prisoners or homeless, and none had resistance results in AST.

Incomplete treatment for TB and coinfection by HIV are frequently discussed factors in published scientific literature concerning the development of resistance. The lack of therapy can select resistant lineages.^( 5 , 8 , 9 )^ Individuals with HIV are more likely to fail and present adverse reactions from different preconized medications schemes, including those associated with TB, which reflects the increase in mortality rates.^( 1 , 9 )^ Studies conducted in the state of Sao Paulo^( 17 )^and Manaus^( 20 )^ indicate statistically significant association between occurrence of resistant/multi-resistant TB and diagnosis of HIV, whereas, in Santa Catarina,^( 18 )^ this relation was not observed. Prevalences of TB/HIV observed in these three studies ranged from 12.3% to 19.4%, therefore corroborating with our study (15.4%).

Another risk factor to be considered is related to continuous use of tobacco products, alcohol and/or illicit drugs. The habit and forms of use of these drugs can favor transmission of bacillus or constitute barriers for the appropriate treatment .^( 4 , 8 , 27 )^

In the state of Sao Paulo^( 17 )^ 28.1%, 20.9% and 16.9% of individuals with TB were, alcoholics smokers, and drug users, respectively. However, these conditions were not correlated with anti-tuberculosis drug resistance. Alcoholism is also not related to multiresistant TB in studies in Manaus^( 20 )^ and Santa Catarina,^( 18 )^ but the use of illicit drugs, observed in this latter study, presented statistically significant correlation with cases of multi-resistant TB. Considering the high use of tobacco products, alcohol, and other drugs by the analyzed population in our article (60.6%), we believe that there is a need of applying specific health policies for this group.

The occurrence of resistance in patients who received care in the hospital where this study was conducted did not present statistically significant with sociodemographic and clinical features collected among individuals. Our sample may be considered small and, for this reason, we emphasize the need of continuing monitoring and further research for longer periods. Studies that consider data from medical records can present limitations, especially because of chance of incomplete information, errors in diagnostic/classification or during recording in the system, low representativeness of some population and restricted analysis of information in the form in which they appear in the system.^( 10 )^ It is importantto keep the team informed on maintaining a complete database to promote better care actions, facilitate on-site communication among different professionals and, perhaps, allow future studies with reduced bias.

## CONCLUSION

The *Hospital Regional Joao Penido,* located in Zona da Mata region, Minas Gerais, is considered a referral center for treatment of tuberculosis, and represents an important site for studies on tuberculosis. The prevalence of resistant tuberculosis were monoresistant, multiresistant and poliresistant cases. The occurrence of anti-tuberculosis resistance drugs did not present statistically significant association with none sociodemographic or clinical features of patients. Findings of the study include relevant information for planning, monitoring, and strategic execution to regional diseases control.
