# Supplementary material for: Analysis of anti-tuberculosis drug resistance and sociodemographic and clinical aspects of patients admitted in a referral hospital
Source: Einstein (Sao Paulo). 2019 Oct 22;18:eAO4620. doi: 10.31744/einstein_journal/2020AO4620 (PMC6896654; doi:10.31744/einstein_journal/2020AO4620)
Supplement: Translation

# Translation: Análise da resistência a medicamentos antituberculose e aspectos sociodemográficos e clínicos de pacientes atendidos em hospital referência

**Authors:** Camilla Resende Bonin, Romário Costa Fochat, Isabel Cristina Gonçalves Leite, Thamiris Vilela Pereira, Marina de Oliveira Fajardo, Carmen Perches Gomide Pinto, Raquel Leite Macedo, Marcio Roberto Silva, Pillar Pace Lacerda Menezes, Nilma Maria José Mendes de Araújo, Ronaldo Rodrigues da Costa

**Affiliations:** 1 Universidade Federal de Juiz de Fora, Juiz de Fora, MG, Brasil.; 2 Hospital Universitário, Universidade Federal de Juiz de Fora, Juiz de Fora, MG, Brasil.; 3 Empresa Brasileira de Pesquisa Agropecuária, Brasília, DF, Brasil.; 4 Fundação Hospitalar do Estado de Minas Gerais, Belo Horizonte, MG, Brasil.

**Keywords:** Tuberculose, Tuberculose resistente a múltiplos medicamentos, Mycobacterium tuberculosis, HIV, Epidemiologia

## Abstract

**Objetivo:**

Verificar a ocorrência de resistência a fármacos antituberculose e a associação com características sociodemográficas e clínicas de pacientes de um hospital referência.

**Métodos:**

Estudo transversal, com dados de pacientes que tiveram a cultura de micobactérias identificada e o respectivo perfil de sensibilidade aos antimicrobianos definido (junho de 2014 a fevereiro de 2016). Foram realizados a análise estatística descritiva e o teste exato de Fisher, para comparação de proporções.

**Resultados:**

O estudo envolveu 104 pacientes, e todos tiveram resultados para *Mycobacterium tuberculosis* . A baciloscopia atingiu alta positividade (93,3%), e 15 pacientes (14,4%) apresentaram linhagens resistentes, sendo 6 (5,6%) multirresistentes. As características sociodemográficas e clínicas não foram associadas à resistência.

**Conclusão:**

A pesquisa permitiu conhecer melhor o perfil dos pacientes com tuberculose e constitui ferramenta para elaboração de políticas públicas específicas. Os pacientes diagnosticados com tuberculose resistente devem ser submetidos à maior supervisão.

## INTRODUÇÃO

A tuberculose (TB) é uma doença infecciosa antiga e reconhecida mundialmente como um problema de saúde pública, sendo um desafio a ser enfrentado por todos os países.^( 1 , 2 )^Associando o diagnóstico no tempo correto com o tratamento adequado, quase todas as pessoas com TB podem ser curadas. No entanto, a infecção causada pelo bacilo *Mycobacterium tuberculosis* constitui uma das maiores causas de morte por doenças infecciosas em adultos. Em 2016, segundo estimativas da *World Health Organization* (WHO), houve 10,4 milhões de novos casos ao redor do mundo e, aproximadamente, 1,6 milhão de mortes.^( 1 )^

O Brasil é considerado um país prioritário pela OMS, exercendo grande impacto no cenário global da doença (82.676 casos notificados em 2016, com incidência de 42,0/100 mil habitantes e taxa de mortalidade de 3,5 óbitos/100 mil habitantes).^( 1 )^Apesar de ter alcançado as proposições das metas de desenvolvimento do milênio (incidência decrescente de TB até 2015 e queda de 50,0% das taxas de prevalência e mortalidade comparadas com as de 1990),^( 3 )^o Brasil tem enormes desafios a serem superados, a fim de atender as novas estratégias para erradicação da TB até 2050.^( 4 )^A agenda continua com as demandas relacionadas a prevenção, diagnóstico precoce com efetividade e adesão ao tratamento, além do problema emergente do aumento de resistência aos fármacos disponíveis.^( 1 , 5 )^

O Sistema de Informação de Tratamentos Especiais de Tuberculose (SITE-TB) é a principal ferramenta da vigilância de casos de TB que necessitam de tratamentos especiais no Brasil, entre eles os casos de TB drogarresistente. O medicamento para o tratamento de TB resistente é liberado nos centros de referência quando o caso é validado no SITE-TB. Segundo informações coletadas no Sistema de Informação de Agravos de Notificação (SINAN), em 2016, foram disgnosticados, no Brasil, 1.044 casos com resistência a medicamentos para TB, ao passo que, no SITE-TB, foram inseridos, neste mesmo ano, 752 casos novos de TB resistente. Essa informação, mesmo com as limitações inerentes aos sistemas, é preocupante, pois sugere que muitos pacientes não realizam o seguimento adequado.^( 6 )^

O controle e a gestão dos programas de combate à TB resistente são mais difíceis comparados com os direcionados aos pacientes que apresentam sensibilidade ao tratamento.^( 7 )^Fatores de risco, como desnutrição, tabagismo, infecção pelo Vírus da Imunodeficiência Humana (HIV)/Síndrome da Imunodeficiência Adquirida (AIDS), *diabetes mellitus* , ambientes pouco ventilados e densamente ocupados são constantemente apontados na literatura como responsáveis pela manutenção e pelo agravamento da TB.^( 1 , 4 , 5 , 8 - 10 )^

As características epidemiológicas e o controle da doença variam de acordo com a região geográfica e sofrem influências de fatores socieconômicos, educacionais e políticos. Na Zona da Mata Mineira, Juiz de Fora (MG) é considerada uma cidade-polo e município estratégico de combate à TB.^( 11 , 12 )^Identificar os casos de TB resistente e conhecer o perfil dos indivíduos envolvidos pode fornecer uma base de evidências para maior manejo clínico do paciente e elaboração de políticas públicas mais efetivas.^( 13 )^Além disso, expandir pesquisas relacionadas à TB é um dos pilares destacados pela OMS para atingir as novas metas de erradicação da doença.^( 14 )^

## OBJETIVO

Determiar a prevalência de resistência a fármacos antituberculose entre pacientes com este diagnóstico em um hospital; investigar a associação dessa condição com características sociodemográficas e clínicas desses pacientes.

## MÉTODOS

O Hospital Regional João Penido (HRJP) está localizado na região da Zona da Mata Mineira, no município de Juiz de Fora. É um hospital público e presta serviços a usuários do Sistema Único de Saúde (SUS) em níveis secundários e terciários de atenção à saúde. Atende toda a macrorregião Sudeste, beneficiando aproximadamente uma população de 1,5 milhão de pessoas. O HRJP possui pronto atendimento e ambulatórios para consultas eletivas nas mais diversas especialidades; no momento da pesquisa, funcionava com a capacidade de atendimento terciário de 212 leitos. A unidade é considerada referência no tratamento da TB.^( 15 )^

Trata-se de estudo transversal, realizado a partir de dados coletados no Sistema Integrado de Gestão Hospitalar (SIGH), sistema de informação integrado em rede utilizado pelo HRJP, o qual permite que o profissional de saúde da Fundação Hospitalar do Estado de Minas Gerais (FHEMIG), que integra o HRJP, acessar o cadastro do usuário, as informações clínicas e os procedimentos e exames anteriormente realizados em qualquer unidade da fundação.

Foi realizada uma análise retrospectiva de laudos do Laboratório de Análises Clínicas do HRJP emitidos entre os meses de junho de 2014 a fevereiro de 2016. Como critérios de inclusão, foram considerados todos os pacientes com TB, independentemente da idade, que possuíam cultura para micobactérias positiva, identificação da espécie e perfil de sensibilidade aos antimicrobianos preconizados.

Os pacientes que tiveram amostras contaminadas ou resultados inconclusivos para espécie ou teste de sensibilidade aos antimicrobianos (TSA) não foram considerados. As culturas positivas de micobactérias isoladas no HRJP foram encaminhadas à Fundação Ezequiel Dias, onde se processaram a identificação e o TSA delas. Os fármacos testados foram estreptomicina (S), isoniazida (H), rifampicina (R), etambutol (E) e pirazinamida (Z).

As interpretações dos resultados do TSA foram realizadas de acordo com as recomendações da WHO, compiladas pelo Ministério da Saúde: monorresistência (resistência apenas a um fármaco); multirresistência (resistência simultânea pelo menos a R e a H); polirresistência (resistência a dois ou mais fármacos, exceto associação do anterior) e resistência extensiva (resistência à R e à H e, simultaneamente, a fluoroquinolona e a um medicamento injetável de segunda linha).^( 16 )^

As informações referentes a sexo, idade, tipo de moradia, resultado de baciloscopia (coloração de Ziehl-Neelsen), forma clínica da TB, coinfecção pelo HIV, presença de anemia, uso de tabaco, álcool e drogas ilícitas dos indivíduos também foram obtidas pelo SIGH, sendo os dados conferidos por dois avaliadores em momentos diferentes.

Em caso da possibilidade de um resultado de cultura sensível e outro resistente (diferentes amostras) para o mesmo paciente, foi priorizada a situação de resistência para categorizar o indivíduo. A correlação destes dados com a ocorrência de casos de resistência foi estabelecida utilizando o teste exato de Fisher, sendo fixado o nível de significância de 0,05. Todos os cálculos, incluindo a análise estatística descritiva, foram realizados com o auxílio do software (SPSS), versão 14.0.

O protocolo do presente estudo foi aprovado pelo Comitê de Ética em Pesquisa com Seres Humanos da FHEMIG, parecer número 732.595/2014, CAAE: 34039214.5.0000.5119.

## RESULTADOS

Foram estudados 104 pacientes, que, no período analisado, tiveram resultados positivos para cultura de micobactérias, identificação da espécie do bacilo e respectiva caracterização do perfil de resistência. Essa amostra foi, predominantemente, composta por indivíduos do sexo masculino (79; 76,0%). A idade dos participantes variou de 17 a 77 anos (média±desvio padrão de 40,2±15,2 anos), sendo que a maior parte encontrava-se na faixa etária mais jovem, de 15 a 44 anos (63; 60,6%).

O diagnóstico clínico de TB pulmonar foi apresentado por quase todos os indivíduos (100; 96,2%), sendo verificados apenas dois casos (1,9%) de TB miliar, um (1,0%) de TB ganglionar e um (1,0%) de TB pleural. A baciloscopia atingiu alto índice de positividade para essa população (97; 93,3%). A espécie de bacilo isolada em todas as culturas foi *Mycobacterium tuberculosis* , sendo reportados 15 casos (14,4%) de alguma resistência aos fármacos utilizados no tratamento da doença. Em relação à classificação, foram observados cinco resultados (4,9%) de monorresistência, seis (5,6%) de multirresistência e quatro (3,9%) de polirresistência ( Tabela 1 ).

**Tabela 1:** Resultados do teste de sensibilidade a antimicrobianos de micobactérias isoladas de pacientes disgnosticados com tuberculose

Teste	n (%)
Teste de sensibilidade a antimicrobianos	
Sensível	89 (85,6)
Resistente	15 (14,4)
Monorresistência	
Estreptomicina	3 (2,9)
Pirazinamida	1 (1,0)
Isoniazida	1 (1,0)
Multirrestitência	
Isoniazida + rifampicina	6 (5,6)
Polirresistência	
Isoniazida + estreptomicina	3 (2,9)
Isoniazida + estreptomicina + etambutol	1 (1,0)

Para essa amostra, não foi possível estabelecer relação estatisticamente significativa entre a ocorrência de resistência a fármacos antituberculose e as características sociodemográficas e clínicas disponíveis dos pacientes ( Tabela 2 ). No entanto, dentre os casos de resistência, as maiores frequências ocorreram em indivíduos do sexo masculino (11; 73,3%), da faixa etária de 15 a 44 anos (10; 66,7%), residentes em casa/apartamento (10; 66,7%), diagnosticados com TB pulmonar (14; 93,3%), HIV negativo (9; 60,0%), com anemia (10; 66,7%) e que relataram uso de álcool, tabaco ou outras drogas (10; 66,7%).

**Tabela 2:** Distribuição dos dados sociodemográficos e clínicos dos pacientes diagnosticados com tuberculose em relação ao perfil de sensibilidade a fármacos antituberculose

Características	Sensível	Resistente	Valor de p^‡^	Total
	n (%)*	n (%)^†^	n (%)	n (%)
Sexo			0,511	
Masculino	68 (76,4)	11 (73,3)		79 (76,0)
Feminino	21 (23,6)	4 (26,7)		25 (24,0)
Faixa etária (anos)			0,413	
15-44	53 (59,5)	10 (66,7)		63 (60,6)
>45	36 (40,5)	5 (33,3)		41 (39,4)
Tipo de moradia			0,449	
Casa/apartamento	69 (77,5)	10 (66,7)		79 (76,0)
Presídio/situação de rua	15 (16,9)	3 (20,0)		18 (17,3)
Não informado	5 (5,6)	2 (13,3)		7 (6,7)
Forma clínica			0,469	
Pulmonar	86 (96,6)	14 (93,3)		100 (96,2)
Extrapulmonar	3 (3,4)	1 (6,7)		4 (3,8)
HIV			0,635	
Sim	13 (14,6)	3 (20,0)		16 (15,4)
Não	50 (56,2)	9 (60,0)		59 (56,7)
Não informado	26 (29,2)	3 (20,0)		29 (27,9)
Anemia			0,466	
Sim	41 (46,0)	10 (66,7)		51 (49,0)
Não	39 (43,8)	4 (26,6)		43 (41,3)
Não informado	9 (10,2)	1 (6,7)		10 (9,6)
Uso de tabaco, álcool e outras drogas			0,147	
Sim	53 (59,6)	10 (66,7)		63 (60,6)
Não	33 (37,1)	3 (20,0)		36 (34,6)
Não informado	3 (3,3)	2 (13,3)		5 (4,8)

Fármacos testados: estreptomicina, isoniazida, rifampicina, etambutol e pirazinamida.^*^Grupo de indivíduos (n=89) com tuberculose não resistente;^†^grupo de indivíduos (n=15) com tuberculose resistente;^‡^teste exato de Fisher com nível de significância de 0,05.

## DISCUSSÃO

Este estudo registrou alta prevalência de resistência geral a fármacos antituberculose (14,4%), sendo 4,9% de monorresistência, 5,6% de multirresistência e 3,9% de polirresistência. A resistência geral apresentada foi ligeiramente superior às apresentadas nas pesquisas desenvolvidas na região norte de São Paulo^( 17 )^e em Santa Catarina.^( 18 )^Neste último, foram analisadas 406 culturas, incluindo 48 (11,8%) isolados resistentes, enquanto a pesquisa no Estado de São Paulo identificou 348 culturas, das quais 31 (8,9%) apresentaram algum tipo de resistência. O perfil de resistência descrito no Estado de São Paulo revelou a predominância de monorresistência (23/31), ao passo que, em Santa Catarina, 23/48 culturas eram multirresistentes. No HRJP, os casos de TB resistente estão bem distribuídos dentro da classificação da OMS, com ligeira predominância de TB multirresistente (6/15).

O monitoramento de micobactérias resistentes, destacado no relatório *End TB Strategy* ,^( 14 )^é uma das ações estratégicas estabelecidas para reduzir a mortalidade e a incidência de TB em todos os países. Estudos populacionais têm sido conduzidos em diferentes locais para melhor compreender a ocorrência dessa situação.^( 13 , 17 - 22 )^A Atenção Primária é o nível ideal para a detecção precoce da doença, mas cuidados de saúde terciários podem ser o ponto de entrada mais comum para pacientes no sistema.^( 17 )^O HRJP, por ser um centro de referência para o tratamento de TB de uma extensa área geográfica (macrorregião Sudeste, a qual abrange aproximadamente uma população de 1,5 milhão de pessoas),^( 15 )^permite estudos epidemiológicos de impactos no cenário local e nacional.

O atendimento à população no HRJP pode ser hospitalar, para os casos que precisam de atentimento mais urgente; ou ambulatorial, para casos que podem ser tratados por meio de consultas periódicas. O hospital atende uma população heterogênea, recebendo pacientes com suspeita ou com complicações clínicas relacionadas à TB, incluindo grupos de risco, como indivíduos em situação de rua, privação da liberdade e/ou usuários de drogas. Todos os pacientes com suspeita de TB pulmonar são submetidos à baciloscopia de escarro e à cultura para micobactéria com teste de sensibilidade. A possibilidade de o hospital receber os casos mais graves ou aqueles com falência de tratamento pode superestimar a prevalência de TB resistente.

A predominância do sexo masculino na presente pesquisa (76,0%) corrobora os dados internacionais e nacionais.^( 1 , 10 , 23 - 25 )^Viana et al.,^( 10 )^realizaram um estudo nacional epidemiológico descritivo de base populacional, considerando todas as notificações no SINAN entre janeiro de 2008 a dezembro de 2011. Esses autores constataram 278.674 novos casos relatados no período analisado e maior prevalência dos homens (65,7%). Outros estudos comprovaram que a prevalência masculina em relação à TB encontra-se entre 65,0 a 85,0% nas diferentes regiões do Brasil.^( 17 , 19 , 23 - 25 )^As pesquisas realizadas nos Estados de São Paulo,^( 17 )^Goiás^( 19 )^e Amazonas^( 20 )^observaram que não houve associação significativa entre ocorrência de TB resistente e multirresistente com sexo.

Da população estudada, 60,6% encontravam-se na faixa etária de 15 a 44 anos. Este resultado também coincide com os estudos de Viana et al.,^( 10 )^(53,9% dos casos notificados eram indivíduos de 20 a 44 anos) e outros.^( 17 , 19 , 23 , 24 )^A faixa economicamente ativa da população é constantemente relacionada à TB, impactando na gestão dos gastos públicos.^( 10 , 23 - 25 )^O paciente com TB resistente aumenta os custos dos serviços de saúde e a sobrecarga na economia, uma vez que o paciente possui prognóstico diminuído, requer maior tempo de tratamento, ocorrem mais efeitos colaterais e tóxicos da medicação e há necessidade de maior controle para evitar a transmissão.^( 20 , 21 , 26 )^Deve-se mencionar que TB resistente também não foi associada com alguma faixa etária específica nos trabalhos de Pedro et al.,^( 17 )^e de Santos et al.,^( 19 )^

Estudos prévios relacionam a forma pulmonar da TB à maior probabilidade de resistência a fármacos. Na TB extrapulmonar, o número de bacilos é menor comparado ao verificado na cavidade pulmonar, portanto, as chances da existência de bacilos resistentes antes do tratamento são menores.^( 23 , 27 )^No trabalho de Pedro et al.,^( 17 )^a forma pulmonar predominou na população (96,2% dos casos), porém não houve relação significativa com a ocorrência de resistência, como também foi verificado para os pacientes do HRJP (96,2% de TB pulmonar).

A TB pulmonar exige maior prioridade em relação às ações de controle, por se tratar da forma clínica transmissível. Nesse sentido, o tratamento e o rompimento do ciclo de transmissão, para serem efetivos, demandam diagnóstico preciso e rápido.^( 1 , 4 , 8 )^A baciloscopia continua a ser um método de escolha, principalmente em países em desenvolvimento, por ser simples, rápido, de baixo custo e, quando executado corretamente, permite identificar mais de 70,0% dos casos de TB pulmonar.^( 28 )^A baciloscopia realizada para os pacientes atendidos no HRJP atingiu elevada positividade (93,3% das amostras), permitindo diagnósticos mais precoces e maior segurança para o controle da doença, além de indicar um padrão de qualidade do serviço e de orientações ao paciente no momento da coleta.

A via de transmissão da TB é aérea, de modo que locais fechados e/ou em condições de higiene reduzidas são favoráveis à continuidade da doença. A população carcerária e os sujeitos que vivem em grandes aglomerados ou em situação de rua são considerados grupos de risco para o desenvolvimento da doença.^( 8 , 10 , 22 , 29 )^A incidência de TB em presídios pode exceder 20 vezes à registrada para a população geral.^( 29 )^Já para os indivíduos em situação de rua, acredita-se em um índice 50 a 60 vezes maior.^( 30 )^O presente estudo, por compreender a demanda atendida pelo HRJP e não se limitar a grupos específicos, apresenta maior limitação para comparar com dados da literatura, pois existem poucos trabalhos com o mesmo modelo metodológico. Vale ressaltar, contudo, que Pedro et al.,^( 17 )^verificaram, no Estado de São Paulo, que a resistência a fármacos antituberculose foi menor em indivíduos em privação da liberdade, com diferença estatisticamente significativa, comparada às pessoas em liberdade. No estudo de Santos et al., que considerou o Estado de Goiás,^( 19 )^sete indivíduos (5,3% da população amostral) encontravam-se em prisões ou em situação de rua, e nenhum teve resultados de resistência no TSA.

O tratamento de TB não concluído e a coinfecção pelo HIV são fatores frequentemente discutidos na literatura científica com relação ao desenvolvimento da resistência. A falha na terapêutica pode selecionar linhagens resistentes.^( 5 , 8 , 9 )^Os indivíduos com HIV são mais propensos às falhas e às reações adversas dos diferentes esquemas medicamentosos preconizados, inclusive quando associados à TB, o que reflete no aumento das taxas de mortalidade.^( 1 , 9 )^Os estudos realizados no Estado de São Paulo^( 17 )^e em Manaus^( 20 )^indicaram relação estatisticamente significativa entre ocorrência de TB resistente/multirressistente e o diagnóstico de HIV, ao passo que, em Santa Catarina,^( 18 )^essa relação não foi comprovada. As prevalências TB/HIV verificadas nestes três estudos variaram de 12,3% a 19,4%, coincidindo com os achados da presente pesquisa (15,4%).

Outro fator de risco a ser considerado refere-se ao uso constante de tabaco, álcool e/ou drogas ilícitas. Os hábitos e as formas de uso dessas drogas podem favorecer a transmissão do bacilo ou constituírem barreiras para o tratamento adequado.^( 4 , 8 , 27 )^No Estado de São Paulo,^( 17 )^28,1%, 20,9% e 16,9% dos indivíduos com TB eram, respectivamente, etilistas, tabagistas e usuários de drogas − condições não relacionadas à ocorrência de resistência aos fármacos antituberculose. O alcoolismo também não foi relacionado à TB multirresistente em Manaus^( 20 )^e em Santa Catarina,^( 18 )^mas o uso de drogas ilícitas, verificado neste último estudo, apresentou relação estatisticamente significativa com os casos de TB multirresistente. Visto o elevado consumo de tabaco, álcool e outras drogas pela população analisada no presente artigo (60,6%), identifica-se a necessidade de aplicação de uma política de saúde específica para esse grupo.

A ocorrência de resistência verificada nos pacientes atendidos no HRJP não apresentou relação estatisticamente significativa com as características sociodemográficos e clínicas obtidas dos indivíduos. A amostra pode ser considerada ainda pequena e, por isso, ressalta-se a necessidade do constante monitoramente e da continuidade da pesquisa por um período maior. Estudos que levam em consideração dados de prontuários podem apresentar algumas limitações, decorrentes, principalmente, do não preenchimento de todas as informações, possibilidade de erro de diagnóstico/classificação ou de registro no sistema, baixa representatividade de alguns segmentos populacionais e análise restrita à forma na qual os dados aparecem no sistema.^( 10 )^É importante que a equipe seja orientada a manter um banco de dados sempre completo, a fim de aperfeiçoar o atendimento, facilitar a comunicação entre os diferentes profissionais do local e, possivelmente, permitir futuras pesquisas científicas, com menores chances de viés.

## CONCLUSÃO

O Hospital Regional João Penido, no âmbito da Zona da Mata Mineira, apresenta-se como um centro de referência no tratamento da tuberculose, compreendendo um importante local de estudo sobre essa doença. A prevalência de tuberculose resistente apresentou casos de monorresistência, multirresistência e de polirresistência. A ocorrência de resistência a fármacos antituberculose não apresentou relação estatisticamente significativa com alguma característica sociodemográfica ou clínica dos pacientes. Os achados do presente estudo compreendem informações relevantes para o planejamento, o monitoramento e a execução de estratégias para controle da doença na região.
